# Bulimia nervosa and treatment-related disparities: a review

**DOI:** 10.3389/fpsyg.2024.1386347

**Published:** 2024-08-14

**Authors:** Kim Wilson, Robert Kagabo

**Affiliations:** College of Health Sciences, Utah Tech University, St. George, UT, United States

**Keywords:** bulimia nervosa treatment, bulimia nervosa research, bulimia nervosa treatment-related disparities, bulimia nervosa diagnosis and treatment, bulimia nervosa and clinical trials

## Abstract

**Introduction:**

Bulimia nervosa (BN) is a type of eating disorder disease usually manifesting between adolescence and early adulthood with 12 as median age of onset. BN is characterized by individuals’ episodes of excessive eating of food followed by engaging in unusual compensatory behaviors to control weight gain in BN. Approximately 94% of those with BN never seek or delay treatment. While there are available treatments, some populations do not have access. Left untreated, BN can become severe and lead to other serious comorbidities. This study is a review of randomized controlled trials to explore available treatments and related treatment disparities. The objective of this review was to identify differences among treatment modalities of BN and aide in the further treatment and research of bulimia nervosa.

**Methods:**

This study followed narrative overview guidelines to review BN treatment studies published between 2010 and 2021. The authors used PubMed and PsychInfo databases to search for articles meeting the inclusion criteria. Search terms included phrases such as, BN treatment, BN and clinical trials, and BN and randomized clinical trials.

**Results:**

Most of the reviewed studies had their sample sizes between 80 and 100% female with age range between 18 and 60 years old. Sample sizes were mostly between 80 and 100% white. Treatment practices included both pharmacological and psychosocial interventions, such as cognitive behavioral therapy (CBT) and limited motivational interviewing (MI). Most studies were in outpatient settings.

**Conclusion:**

Reviewed research shows that certain populations face disparities in BN treatment. Generally, individuals older than 60, males and racial minorities are excluded from research. Researchers and practitioners need to include these vulnerable groups to improve BN treatment-related disparities.

## Background

Bulimia nervosa (BN) is a serious eating disorder (ED) that usually manifests between adolescence and early adulthood (Hail and Le Grange, 2018). BN is characterized by episodes of recurrent binge eating (Martins et al., 2020). BN involves engaging in compensatory behaviors such as self-induced vomiting, misuse of laxatives, diuretics, or enemas (Mond, 2013; American Psychiatric Association, 2022). Other compensatory behaviors may include activities such as fasting or excessive exercise (Mond, 2013; American Psychiatric Association, 2022). The episodes can be mixed and are classified as recurrent episodes of binge eating in any 2-h period that is larger than most people would during a similar period (Mond, 2013; American Psychiatric Association, 2022). Individuals might go as far as to arrange their schedules to accommodate episodes of bingeing (Harrington et al., 2015).

The median age of onset is 12.4 (Hail and Le Grange, 2018), however onset could be as high as 18–44 years old with a lifetime prevalence of 1.5% among females, and 0.5% among males (Udo and Grilo, 2018). Other research has shown the range of lifetime prevalence among females to be 0.3–4.6% and 0.1–1.3% among males (van Eeden et al., 2021). Research also shows that 85–94% of those with BN never seek professional help, or delay treatment by 4–5 years (Mathisen et al., 2017). Left untreated, BN can become severe and lead to other comorbidities or bad health outcomes, including death in some cases (Hail and Le Grange, 2018). This study is a review of randomized controlled trials to explore available treatments and related treatment disparities. While different authors may define health disparities in different ways, our definition is one generally accepted which is that health disparities are differences or gaps in health outcomes, or even healthcare access, and treatment among populations (Riley, 2012; Arcaya et al., 2015). These differences are unjust and preventable and can occur between racial groups, social economic status, or gender (Riley, 2012; Arcaya et al., 2015). These differences or gaps would not normally occur if the distribution of resources were fair (Riley, 2012; Arcaya et al., 2015). Our objective was to identify these differences in the treatment and research of bulimia nervosa among populations. An identification of these differences is key in the successful treatment and research of bulimia nervosa.

## Diagnosis

The primary diagnostic criterion of BN is stated in the Diagnostic and Statistical Manual of Mental Disorders Fourth edition, DSM-IV and Fifth edition, DSM-V (MacDonald et al., 2014; Harrington et al., 2015; American Psychiatric Association, 2022). Individuals with BN are usually of normal height and weight (Frank, 2012), but can also be overweight which makes the diagnosis of BN challenging (Harrington et al., 2015). Also, according to the DSM criteria, BN is characterized by an episode of binge eating by (a) eating in a discrete period, an amount of food that is larger than most individuals would eat in a similar period and under similar circumstances, and lacking control of eating during this episode. (b) recurrent compensatory behaviors that prevent weight gain, (c) at least once per week for 3 months, (d) self-evaluation of body shape and weight is unduly influenced, and (e) this behavior is not that of anorexia nervosa (Mond, 2013; Harrington et al., 2015; American Psychiatric Association, 2022). The individual is in partial remission if some, but not all criteria have been met for a sustained period of time and will be considered full remission if none of the criteria is met for a sustained period of time (Harrington et al., 2015; American Psychiatric Association, 2022). The average frequency of binge eating and purging has decreased from twice per week, per the DSM-IV, to once per week (Mond, 2013; Harrington et al., 2015; American Psychiatric Association, 2022), which was the increasing prevalence for BN ranging from 4 to 6.7% (Nitsch et al., 2021).

The severity of BN is based upon the frequency of compensatory behaviors and may reflect other symptoms and functional disability and are categorized based upon average episodes of compensatory behaviors per week. These categories include Mild 1–3, Moderate 4–7, Severe 8–13 and Extreme 14+ (Harrington et al., 2015; Nitsch et al., 2021; American Psychiatric Association, 2022).

## Untreated or undertreated BN

Untreated or undertreated BN is associated with several comorbidities. These comorbidities may include psychiatric disorders, hopelessness, shame and impulsivity, which can contribute to non-suicidal self-harm, suicidal ideation and death by suicide (Nitsch et al., 2021). The suicide rates among individuals with BN are high where compared to the general population, they are 8 times more likely to die by suicide (Preti et al., 2011; Cucchi et al., 2016; Nitsch et al., 2021). The standardized mortality rates among those with BN are elevated at 1.5 to 2.5% (Arcelus et al., 2011; Nitsch et al., 2021). This higher mortality rate in BN is due to the medical complications of purging (Nitsch et al., 2021). These purging behaviors and laxative use can cause electrolyte imbalances leading to increased risk of cardiovascular disease, including ischemic heart disease and in some cases resulting in death in females (Nitsch et al., 2021).

Other problems associated with purging include dental erosion and hypertrophy of salivary glands (Harrington et al., 2015; Nitsch et al., 2021), trauma to the pharynx, increased risk of aspiration pneumonia, irregular menses due to endocrine system disruption and gastrointestinal problems (Nitsch et al., 2021). The binge eating aspect of BN can also cause gastrointestinal problems such as bloating, dysphagia and acid reflux (Nitsch et al., 2021). Prognosis and recovery are variable and there is an increased risk of relapse with psychological dysfunction and body image disturbance. There is evidence to support changes in neuronal activity and suggest a link in BN with body image distortion (Wang et al., 2019). Poor outcomes can be attributed to fewer follow-up years, increased drive for thinness and beginning treatment at an older age (Nitsch et al., 2021). It is however estimated that with proper treatment 80% of individuals with BN achieve remission (Harrington et al., 2015).

## Methods

**Figure 1:**
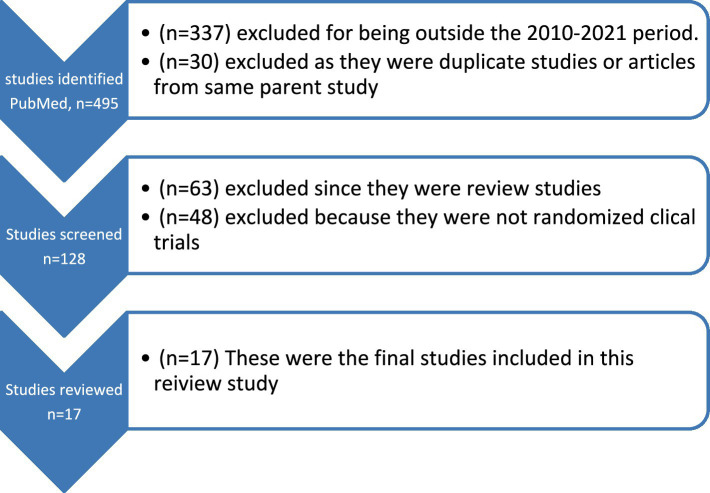
Flow chart of studies reviewed.

This study followed narrative overview guidelines to review randomized controlled trial (Wonderlich et al., 2014; Jacobi et al., 2017; Mannan et al.., 2021) studies published between 2010 and 2021. The authors searched PubMed and PsychInfo databases to search for articles meeting the inclusion criteria. Search terms included phrases such as bulimia nervosa treatment, bulimia nervosa and clinical trials, bulimia nervosa and randomized clinical trials, or bulimia nervosa diagnosis and treatment. Any studies that did not involve randomized controlled trials and treatment of bulimia nervosa were excluded. Review studies such as systematic reviews or meta-analysis were also excluded from review for this study. This paper was not a review of review studies and because such review works had already been completed by other investigators, they were left out of this current review study. Of the 685 studies resulting from the search terms used, 17 studies met the inclusion our inclusion criteria and were reviewed for this study. The variables examined included, sample size, sex, mean age, race/ethnicity, study setting (inpatient/outpatient), intervention/treatment type, and response to interventions (Figure 1).

## Results

Table 1 (Summary of Bulimia Nervosa Randomized Clinical Trials) is a summary of journal articles published between 2010 and 2021 which were reviewed for this study. Following the inclusion criteria, 17 studies were included in this review. Most of the reviewed studies had their sample sizes between 80 and 100% female with age range between 18 and 60 years old. Sample sizes were mostly between 80 and 100% white. Treatment practices included both pharmacological, and behavioral, or psychosocial interventions, such as cognitive behavior therapy (CBT) and limited motivational interviewing (MI), using at-home questionnaires. Most psychosocial interventions used were CBT and incorporated other strategies such as dialectical behavioral therapy (DBT), family-based therapy (FBT), limited MI, guided self-help (GSH), integrative cognitive-affective therapy (ICAT), virtual reality (VR), mindfulness and acceptance-based therapy (MABT), psychodynamic therapy (PDT), identity intervention program (IIP) and supportive psychotherapy (SPT). Other studies included those utilizing physical exercises and dietary therapy (PED-t), transcranial magnetic stimulation (rTMS), direct current stimulation (DCS), and pharmacological treatments using phentermine/topiramate (PHEN/TPM-ER). Most of the studies were done in outpatient settings.

**Table 1:** Summary of bulimia nervosa randomized controlled trial intervention studies (2010–2021).

Author (Year)	Sample Size (*N*)	Sex (%) F/M	Mean Age (Range) (Years)	Race/Ethnicity (%)	Study setting	Intervention treatment (arms)	Response	Other
Chami et al. (2021)	*N* = 78 BN = 40 BED = 38	*F* = 91 *M* = 8	BN =30.6 BED = 36.6 (18–60)	White = 75.6 Black = 6.4 White/Black mix = 2.6 Asian = 6.4 Middle Eastern = 3.9 Latin Am = 3.9	Outpatient (At-home questionnaires)	Training conditions General inhibitory control, *n* = 38 Food specific inhibitory, *n* = 40	Those with BN exhibited higher likelihood of meal skipping, restriction and compensation than BED. Negative affect was higher with BN than BED. Mood was improved on non-binge days vs. binge days for BN and BED.	Inclusion criteria: Body Mass Index (BMI) at least 18.5 Measures: eating disorder examination questionnaire (EDE-Q); Mood and Food Diary; Patient Health Questionnaire −9; Generalized Anxiety Disorder Questionnaire-7
Chen et al. (2017)	*N* = 109 GSH = 42 DBT = 36 CBT + =31	*F* = 100 *M* = 0	GSH =38.6 DBT = 38.2 CBT + =37.8	White =73.4 Afr. Am = 17.4 Asian = 1 Hispanic = 10.1 Other = 8.3	Outpatient (Adult Eating Disorders Program) (Participants resided within commuting distance)	Guided self-help (GSH) Dialectical behavior therapy (DBT) and Cognitive behavioral therapy (CBT)	Objective Binge Days (OBD) lower at end or treatment (EOT) with DBT and CBT, diminished in all groups at 12-months with DBT/CBT+ > cGSH, Secondary outcomes: decr. Vomiting, OBD abstinence, ED psychopathology, BMI and co-occurring Axis 1 disorders.	Where GSH responses were weak, clients were randomized to DBT or CBT+ for 6 months
Ferrer-Garcia et al. (2017)	*N* = 64 BN = 35 BED = 29	*F* = 70.3 *M* = 29.7	Onset BN = 18.06 Onset BED = 24.55	Not specified	Inpatient (Therapist referral after unsuccessful first treatment)	Additional-CBT (*n* = 32) Virtual reality cue exposure therapy (VR-CET) (*n* = 32)	Both treatments showed decreased self-reported purging, desire for thinness uncontrolled overeating. Abstinence of behaviors higher in BN vs. BED with VR-CET	Mean BMI = A-CBT = 29.47 VR-CET = 27.78 Parallel group study. 13 months; 60-min sessions 2/week for 3 weeks. Treatment-resistant patients were included and severe mental disorders were excluded.
Gay et al. (2016)	*N* = 51	F = 100 *M* = 0	rTMS =27 sham rTMS = 29.5 (18–40)	Not specified	Outpatient (Academic centers in France, Montpelier and Saint Etienne)	Repetitive transcranial magnetic stimulation (rTMS) (*n* = 26) sham rTMS (*n* = 25)	No significant differences between groups for binge/purge days.	Inclusion criteria: 6 months with no improvement of Selective Seratonin Reuptake Inhibitor treatment
Hildebrandt et al. (2017)	*N* = 66 CBT-GSH = 33 CBT-GSH + Noom = 33	*F* = 83.3 *M* = 16.7	32.11	Non-White = 37.9 Hispanic or Latino/a = 16.7	Outpatient (Residing within 50 miles of study setting)	CBT-GSH CBT-GHS + Noom smart phone app	Significant change in OBEs favoring CBT-GSH + Noom as compared to CBT-GSH Remission not statistically different. Greater meal/snack adherence in CBT-GSH + Noom as compared to CBT-GSH	Mean BMI 27.53 8 sessions for 12 weeks Assessments at 0, 4, 8, 12, 24, and 36 weeks Excluded: significant medical illness, substance dependence, bipolar, psychosis, psychotropics
Jacobi et al. (2017)	*N* = 253 IN@ = 126 TAU = 127	F = 100 *M* = 0	IN@ = 21.49 TAU = 21.99	Not specified	Inpatient (Psychosomatic hospitals, Germany)	Web-based aftercare short message service (IN@) Treatment as usual (TAU): mixed CBT and psychodynamic CBT	Post Intervention: Binge eating 28% lower than TAU Vomiting 46% in IN@ lower than TAU Compensatory behaviors 35% lower in IN@ than TAU	BMI < 17.5 excluded 16 weeks web-based for 9 months; Inpatient baseline assessments, discharge and 9 months follow up OR, TAU group
Juarascio et al. (2021)	*N* = 44	*F* = 88.6 *M* = 11.4	CBT =35.22 MABT = 29.77 (18–60)	White = 88.9 Black = 5.6 Asian = 5.6	Outpatient (Drexel University IRB)	CBT (*n* = 18) Mindfulness and acceptance based therapy (MABT) (*n* = 26)	BN symptom reduction rates improved in both CBT and MABT Depression and quality of life improved more in MABT than in CBT	Mean BMI: CBT = 27.54 MABT = 25.03 20 OP sessions over 20 weeks Primary Measures: Difficulties in Emotional Regulation Scale; Distress Tolerance Scale; Acceptance and Action Questionnaire-II; Valued Living Questionnaire-II; Secondary Measures: EDE; BDI; Quality of Life Inventory; Clinician Global Impressions Scale
Kekic et al. (2017)	*N* = 39	*F* = 94.9 *M* = 5.1	25.85 (18–48)	White = 74.4 Other = 25.6	Outpatient (Institute of Psychiatry, Psychology and Neuroscience, London, UK)	Transcranial Direct Current Stimulation (tDCS) or sham tDCS Anode/Cathode placements: Anode left/cathode right (AL/CR) and Anode right/cathode left (AR/CL)	Reduced ED cognitions in AR/CL tDCS>AL/CR and sham tDCS Mood improvement after AR/CL compared to sham and AL/CR tDCS. tDCS comparable effects on wanting/liking food and bulimic behaviors after 24-h post-simulation	Mean BMI = 21.65 3 sessions of psychological and neurocognitive measures 24 h post sessions Used Mizes Eating Disorder Cognitions Questionnaire-Revised; Pregnant women were excluded.
LeGrange et al. (2015)	*N* = 130	*F* = 94 *M* = 6	15.8 (12–18)	46 Self-reported from ethnic minorities Other = 54	Outpatient (University of Chicago and Stanford University)	CBT (*n* = 58) Family based therapy (FBT) (*n* = 52) Supportive psychotherapy (SPT) (*n* = 20)	Abstinence Rates: CBT <0.2 FBT <0.4 SPT Inconclusive	18 OP sessions over 6 months Participants had Partial BN, purging at least once per week over 6 weeks, OR Expected Body Weight > 85
MacDonald et al. (2017)	*N* = 44 BN = 81.8% Purging Disorder = 18.2%	*F* = 100 *M* = 0	27.3 (17+)	White = 75 Black = 6.3 Asian = 6.9 Latina = 2.3 Oth = 8.6	Outpatient (Day hospital (DH) treatment)	CBT- Rapid Response (*n* = 23) Motivational Interviewing (MI) (*n* = 21)	Rapid Response: No differences in eating behaviors between groups; Significantly fewer binge/purge with CBT-RR. EOT: Improvements in weight/shape > in CBT-RR; no difference in ED frequency and abstinence between groups. Improvements in depression, emotional regulation in both groups; no change in self-esteem	BMI > =19 Measures: EDE, EDE-Q, Difficulties in Emotional Regulation Scale, BDI-II, RSES 8 weeks. CBT-RR or MI as adjunct to DH treatment. 4 weekly, 1 h individual sessions with 1–2 sessions prior to admittance. Inclusion: no previous admissions to day program in same facility in 5 years. Exclusion: AN, BED, suicidality; psychosis or mania; medical instability
Mannan et al. (2021)	*N* = 100 (50 per arm, 10 per group)	*F* = 96 *M* = 4	40.55 (18+)	Caucasian = 74.5 Other = 25.5	Outpatient (Office-based in Brazil)	CBT-E HAPIFED: Health Approach to Weight Management and Food in Eating Disorders; Body Weight Loss Therapy (BWLT) w/ CBT-Enhanced	HAPIFED not significant to reduce body weight or ED symptoms more than CBT-E	BMI 27> to <40 30 sessions of CBT or BWLT HAPIFED 1 individual and 29 group over 6 months incl baseline, EOT, 6o, 12 months assessments Exclusion: bariatric surgery, weight loss medication, appetite regulation, psychosis or bipolar, ED psychotherapy or suicide risk.
Mathisen et al. (2020)	*N* = 164	*F* = 100 *M* = 0	BN = 27.2 BED = 29 (18–40)	Not specified	Outpatient	CBT (*n* = 78) Physical exercises/dietary therapy (PED-t) (*n* = 78)	Full or partial remission achieved: CBT 30%; PED-t 50%	BMI between 17.5 and 35 BN or BED at least 3 months 16 weeks; 20 group sessions
Safer et al. (2019)	*N* = 22 BED = 18 BN = 4	*F* = 96 *M* = 4	42.9 (18–60)	White = 54.5 Black/Afr. Am. = 13.6 Oth = 31.8, incl. Hispanic	Outpatient	PHEN/TPM-ER (*n* = 12) Placebo (*n* = 10)	OBE days reduced by 7.3 days; abstinence 63% while on PHEN/TPM-ER vs. placebo. No significant difference between groups.	BMI 31.1 34 weeks total; 12 weeks PHEN/ TPM-ER (12) or placebo (10), 2 weeks drug wash-out, 12 weeks crossover
Stefini et al. (2017)	*N* = 81	*F* = 100 *M* = 0	18.7 (14–20)	Not specified	Outpatient (Two research centers in Germany)	CBT (*n* = 39) Psychodynamic therapy (PDT) (*n* = 42)	Remission rates: CBT = 33.3% PDT = 31.0% CBT Binge/purge frequency (*p* < 0.001), and EDE/EDE-Q	60 sessions of CBT or PDT Measures: Baseline 15th session 30 session 45th session
							PDT binge (*p* = 0.01) and purge (*p* = 0.05) frequency CBT remission 38.5% and PDT 31% remission at 12-month No significance between EOT and 12 months follow-up	EOT 12 month-post Exclusion criteria: AN, psychosis, substance abuse, suicidality, Attention Deficit Hyperactivity Disorder and IQ < 80
Stein et al. (2013)	*N* = 69	*F* = 100 *M* = 0	IIP = 23.5 SPI = 24.4 (18–35)	White = 75.36 Other = 24.64	Outpatient (Recruited through provider referrals, near local university/college)	Identity intervention program (IIP) (*n* = 34) Supportive psychotherapy intervention (SPI) (*n* = 35)	Decrease in purging and restricting rates; Increase in positive self-schemas, neither significant between groups	Exclusion criteria: pregnancy, psychotropics 2 weeks prior, psychotherapy. Primary outcome measures: Ecological Momentary Assessment (EMA) of Eating Disorders; Eating Disorder Inventory (EDI)
Valenzuela et al. (2018)	*N* = 110	*F* = 100 *M* = 0	(12–18)	Not specified	Outpatient	CBT-A (*n* = 52) FBT-BN (*n* = 58)	Mean BDI reduction from baseline to EOT: CBT = 24.5%, FBT = 36.9% 12 months follow up: CBT = 46.4% FBT = 46.9% Mean RSES score at baseline: CBT = 21.85 FBT = 23.39 12 months follow-up: CBT = 27.53 FBT = 29.48	10% of participants were taking stable doses of antidepressants, Beck Depression Inventory (BDI) and Rosenberg Self-Esteen Scale (RSES) taken at baseline; session 9; EOT; and 6 and 12 months follow-ups.
Wonderlich et al. (2014)	*N* = 80	*F* = 90 *M* = 10	CBT = 28.8 ICAT = 25.8	White = 87.5 Asian = 6.3 Hisp = 2.5 Afr. Am. = 1.3 Nat. Am = 1.3 Oth = 1.3	Outpatient (Clinical sites from Minnesota and North Dakota)	CBT-E (*n* = 40) Integrative cognitive-affective therapy (ICAT) (*n* = 40)	Abstinence Rates: ICAT: EOT 37.5%, 4 months 32.5% CBT-E: EOT and 4 months 22.5%	BMI 23.9 21, 50-min individual sessions at 2x wk. for 4 weeks, 19 weeks total 4 phases EDE-Q baseline, EOT, 4 months No significant secondary outcomes

## Discussion

Our findings show that several behavioral interventions are used for the treatment of BN, with CBT as the preferred choice (Mathisen et al., 2017; Burmester et al., 2021). Other behavioral interventions included VR, DBT and FBT. Most individuals with BN do not seek treatment and instead present for weight loss issues, making CBT the first line of defense for treating eating disorders (ED) (Donnelly et al., 2018). Other tools such as the Stroop Test guide clinicians in the understanding of the effects of visual stimuli as it translates into emotional, psychological and neural responses in those with ED and therefore aides in forming clinical treatment programs using CBT and visual imagery (Burmester et al., 2021). The vicious cycle of compensatory strategies that affect perceptions of weight and shape can render CBT effective in treating BN.

The focus of CBT-BN can restructure the cognitive distortions of body shape and weight, perfectionism, low self-esteem, interpersonal stress, and mood tolerance. While self-reporting can be a source of shame, it is important in the problem-solving process during CBT-BN treatment (Hagan and Walsh, 2021). However, FBT is shown to be more effective in adolescents than in adults (Gorrell et al., 2019). Physical exercise can also be used to treat BN symptoms, however, it is not typically used in clinics due to the propensity for those with ED to overexercise and the clinicians’ fear that such exercise prescription would increase the compensatory behaviors (Bratland-Sanda et al., 2009; Quesnel et al., 2018; Mathisen et al., 2020). A combination of Dietary Therapy and Physical Exercise (PED-t) was studied as a trial, as a new method of treatment and was hypothesized to re-establish healthy patterns by focusing on the functionality of the body rather than appearance. This also aimed to provide knowledge to those suffering with ED, specifically BN and binge eating disorder to change thought patterns. The concern for this treatment approach was the overall long-term effectiveness yet is still an approach that is being considered as part of their evidenced-based practice (Mathisen et al., 2020).

## CBT and other behavioral interventions compared

Individuals receiving CBT treatment have reported a decreased desire for thinness and demonstrated fewer purging episodes (Ferrer-García et al., 2017). Studies also compared CBT with other methods such as Physical Exercise/Dietary Therapy (PED-t). A comparison of CBT and PED-t found that in addition to reduced depression symptoms, PED-t performed just as good as CBT in reducing the symptoms of BN and binge eating disorder and improving other psychosocial impairment (Mathisen et al., 2020). There were also increased abstinence rates with VR (Ferrer-García et al., 2017), and mindfulness and acceptance-based treatment (MABT) performed just as equal to CBT in increased retention rates and reductions in BN symptoms (Juarascio et al., 2021). Participants receiving Guided Self-Help (GSH) had fewer objective binge eating days (OBD), vomiting, psychopathology and improved abstinence rates (Chen et al., 2017) and greater meal/snack adherence with GSH and CBT combined (Hildebrandt et al., 2017). These results suggest that while CBT is the preferred method, approaches such as PED-t and MABT can be just as good and even good alternatives when use of CBT is not possible (Mathisen et al., 2020; Juarascio et al., 2021). Abstinence rates among adolescents aged 12–18 were greater with FBT (Le Grange et al., 2015), and with improved cognitive symptoms, self-esteem and depressive symptoms as compared to CBT and SPT (Ciao et al., 2015). Further research is needed to determine effective treatment outcomes for adolescents.

In addition to CBT, other innovative methods were found in the review. Identity Intervention Program (IIP) was used to identify self-schemas and promote new cognitive structures. Supportive Psychotherapy Intervention (SPI) was used as the control group for this study. Participants used descriptive cards to identify how they think about themselves, along with the Beck Depression Inventory (BDI), Psychological Well-Being scales and a Health Survey during this study. At end of treatment, the IIP group had higher mean increases in positive self-schemas, with no significance in the SPT group, taken from baseline measurements (Stein et al., 2013). Repetitive transcranial magnetic stimulation (rTMS) and sham rTMS were used to determine the efficacy of reducing food cravings in bulimic patients by stimulating the left dorsolateral prefrontal cortex. This resulted in no significant differences between groups for binge/purge days (Gay et al., 2016). Use of at home questionnaires were used to determine food cravings, binge-eating, negative mood, and meal skipping using general inhibitory control and food specific inhibitory control methods.

Results showed that although negative mood and binge eating co-occurred, there were no significant differences in binge eating days and negative mood between patient groups. Food cravings were also higher on binge days and did not differ between groups (Chami et al., 2021). Other methods to consider are transcranial Direct Current Stimulation (tDCS) which has shown comparable effects of wanting and liking food and bulimic behaviors after receiving tDCS treatment vs. sham tDCS. This method utilized two varieties of anode/cathode placement to determine effects on mood, ED cognitions and bulimic behaviors, exhibiting reduced ED cognitions and mood improvement with anode right/cathode left (AR/CL) versus reduced bulimic behaviors with anode left/cathode right (AL/CR) placement (Kekic et al., 2017).

## Pharmacological treatment

In cases of comorbidities, pharmacological interventions are used. Over half of those diagnosed with BN meet criteria for having a major depressive episode, and others may also suffer from obsessive compulsive disorder, social phobia, anxiety, or substance use disorders. Other psychological traits could be perfectionism, social withdrawal, emotional dysregulation, and poor distress tolerance (Harrington et al., 2015). Due to the possibility of underlying depression or other comorbidities, use of selective serotonin reuptake inhibitors (SSRIs) may be prescribed for BN to decrease binge and purge frequency especially among those who have not responded initially to psychotherapy (Harrington et al., 2015). Use of Bupropion has been contraindicated due to an increased risk of seizure and has a boxed warning (Nitsch et al., 2021). Use of stimulant medications are often discontinued until individuals have been abstinent of purging behaviors for a period of time (Nitsch et al., 2021). Research shows that among adolescents with BN, 88% meet the criteria for one or more comorbidities for mood and anxiety disorders, and low self-esteem, with depressive disorder being the most common.

Although both CBT and FBT have shown to improve depression and self-esteem for adolescents 12–18, based on the Beck Depression Inventory and Rosenberg Self-Esteem Scale (RSES), neither treatment is adequate and a supplemental pharmacological intervention is helpful especially among individuals with BN (Valenzuela et al., 2018).

## Gaps

This study identified some treatment-related disparities of individuals with BN. Most of the studies reviewed enrolled participants between age 18 and 60 and therefore creating a gap of limited research on those aged under 12 and over 60 (Safer et al., 2019; Stedal et al., 2023). Out of the 17 studies we reviewed, only 3 included participants between ages 12 and 18 (Le Grange et al., 2015; Stefini et al., 2017; Valenzuela et al., 2018). While the majority onset age is between 18–20 and 30–44, there is evidence that individuals under 18 and over 44 also suffer from BN (Kotler et al., 2003; Silén and Keski-Rahkonen, 2022; Cadwallader et al., 2023). Along with these age groups, males are often excluded from the research and interventions, yet it is known that males also suffer from BN (Ferrer-García et al., 2017; Hildebrandt et al., 2017) and females’ responses to interventions could be different from that of males. Also unknown, and mostly excluded in the literature is how BN affects other marginalized populations such as LGBTQ individuals (Simone et al., 2020). Moreover, many studies also exclude individuals that are not identified as White/Caucasian (Stein et al., 2013; Chami et al., 2021; Juarascio et al., 2021) and further research is needed for non-English speaking individuals and/or less acculturated minoritized groups. Individuals with serious mental illness and severe substance use are generally excluded from studies. Most of the studies in this review were done in outpatient settings (Gay et al., 2016; Chen et al., 2017) leaving few opportunities or none for individuals in inpatient settings to participate in research and newer treatment opportunities. Interventions presented in this review are largely behavioral and research is needed to determine the impact of psychotropics and holistic medicine alone, or in conjunction with behavioral therapies.

## Conclusion

Research shows that all in the general population face risks of developing BN and that treatments are available. However, in research reviewed in this study, certain populations such as those of male sex, age group, or racial minority groups are excluded from research and treatment options and therefore creating bulimia nervosa treatment-related disparities. To improve and eliminate the bulimia nervosa treatment disparities, researchers and practitioners need to include marginalized populations such as the LGBTQ or other vulnerable groups such as racial minorities, and all those populations generally left out of research and treatment.

## Author contributions

KW: Conceptualization, Investigation, Methodology, Writing – original draft, Writing – review & editing. RK: Supervision, Writing – review & editing.

## Glossary

AL/CR: Anode left/cathode right
AN: Anorexia nervosa
AR/CL: Anode right/cathode left
BDI: Beck depression inventory
BED: Binge eating disorder
BMI: Body mass index
BN: Bulimia nervosa
CBT: Cognitive behavioral therapy
DBT: Dialectical behavioral therapy
DSM: Diagnostic and statistical manual of mental disorders
ED: Eating disorder
EDE-Q: Eating disorder examination-questionnaire
EOT: End of treatment
FBT: Family based therapy
GSH/cGSH: Guided self help/with CBT
ICAT: Integrative cognitive-affective therapy
IN@: Web-based aftercare
IIP: Identity intervention program
MABT: Mindfulness and acceptance-based treatments
MI: Motivational interviewing
OBE/D: Objective binge eating/days
OP: Outpatient
PDT: Psychodynamic therapy
PED-t: Physical exercise/dietary therapy
PHEN/TPM-ER: Phentermine/topiramate ER
rTMS: Repetitive transcranial magnetic stimulation
RSES: Rosenberg self-esteem scale
SPI/SPT: Supportive pshychotherapy intervention/treatment
tDCS: Transcranial direct current stimulation
VR-CET: Virtual reality cue exposure therapy
